# Preexisting Cardiovascular Disease, Hypertension, and Mortality in Peritoneal Dialysis

**DOI:** 10.31083/j.rcm2401030

**Published:** 2023-01-16

**Authors:** Juan Wu, Xiaojiang Zhan, Yueqiang Wen, Xiaoyang Wang, Xiaoran Feng, Fenfen Peng, Niansong Wang, Xianfeng Wu, Junnan Wu

**Affiliations:** ^1^Urology & Nephrology Center, Department of Nephrology, Zhejiang Provincial People's Hospital (Affiliated People's Hospital, Hangzhou Medical College), 310014 Hangzhou, Zhejiang, China; ^2^Department of Nephrology, the First Affiliated Hospital of Nanchang University, 330209 Nanchang, Jiangxi, China; ^3^Department of Nephrology, the Second Affiliated Hospital of Guangzhou Medical University, 510260 Guangzhou, Guangdong, China; ^4^Department of Nephrology, the First Affiliated Hospital of Zhengzhou University, 450052 Zhengzhou, Henan, China; ^5^Department of Nephrology, Jiujiang No. 1 People's Hospital, 332000 Jiujiang, Jiangxi, China; ^6^Department of Nephrology, Zhujiang Hospital of Southern Medical University, 510280 Guangzhou, Guangdong, China; ^7^Department of Nephrology, Shanghai Jiao Tong University Affiliated Sixth People's Hospital, 200233 Shanghai, China; ^8^Clinical Research Center for Chronic Kidney Disease, Shanghai Jiao Tong University Affiliated Sixth People's Hospital, 200233 Shanghai, China; ^9^Department of Nephrology, Zhejiang University Medical College Affiliated Sir Run Run Shaw Hospital, 310016 Hangzhou, Zhejiang, China

**Keywords:** peritoneal dialysis, mortality, cardiovascular disease, hypertension, prognosis

## Abstract

**Background:**

Preexisting cardiovascular disease (CVD) and hypertension are each associated with poor prognosis in peritoneal dialysis (PD) patients. Joint associations of preexisting CVD and hypertension have not been comprehensively evaluated in this population.

**Methods:**

We conducted a retrospective cohort study of 3073 Chinese incident PD patients from five dialysis centres between January 1, 2005, and December 31, 2018. The joint associations between preexisting CVD, hypertension, and mortality were analysed using Cox regression models.

**Results:**

Over a median of 33.7 months of follow-up, 581 (18.6%) patients died, with 286 (9.3%) deaths due to CVD. After adjusting for confounding factors, the preexisting CVD coexisting with hypertension, preexisting CVD, and hypertension groups had higher risks of all-cause mortality (hazard ratio [HR]: 3.97, 95% confidence interval [CI]: 3.06 to 5.15; HR: 2.21, 95% CI: 1.29 to 3.79; and HR: 1.83, 95% CI: 1.47 to 2.29, respectively) and CVD mortality (HR: 4.68, 95% CI: 3.27 to 6.69; HR: 2.10, 95% CI: 0.95 to 4.62; and HR: 1.86, 95% CI: 1.36 to 2.54, respectively) than the control group without preexisting CVD or hypertension (*p* for trend <0.001). There was no interaction between subgroup analyses (*p *> 0.05). The joint associations showed similar patterns using the Fine–Gray competing risk models.

**Conclusions:**

Preexisting CVD and hypertension at the start of PD were additive prognostic utilities for mortality, and preexisting CVD was more strongly associated with mortality than hypertension.

## 1. Introduction

The overall prognosis of end-stage renal disease (ESRD) is poor, with only 11% of peritoneal dialysis (PD) patients surviving past 10 years [1]. Cardiovascular disease (CVD) accounts for approximately 40% of deaths in dialysis patients [2]. PD patients have 10 to 30 times higher CVD mortality than the general population even after adjusting for age, sex, and ethnicity [3], and they also have a high prevalence of traditional CVD risk factors, such as preexisting CVD, hypertension, and diabetes mellitus. Managing CVD risk factors is a priority in dialysis patient management.

PD patients with preexisting CVD have poorer survival than those without preexisting CVD [4, 5]. Two larger PD facilities in China separately reported that the presence of hypertension affects 65.7% and 73.8% of patients, with 30.3% and 10.5% prevalence of preexisting CVD, respectively [6, 7]. Another national dialysis study of the United States reported a 72.6% prevalence of hypertension, with a 25.9% prevalence of coronary artery disease [4]. Previous studies reported that elevated, lower, or uncontrolled blood pressure (BP) is associated with increased mortality in the dialysis population [8, 9, 10]. However, no study has evaluated preexisting CVD and hypertension simultaneously in dialysis patients, and the joint association of preexisting CVD and hypertension with mortality in PD patients has not been evaluated in this setting. Our aim was to assess whether preexisting CVD would provide additive prognostic information to hypertension and to compare the strength of the association with mortality with that of hypertension in patients on continuous ambulatory peritoneal dialysis (CAPD).

## 2. Materials and Methods

### 2.1 Study Design and Population

We conducted a retrospective cohort study of 3073 incident CAPD patients from five PD centres in three provinces in China (The First Affiliated Hospital of Nanchang University, Nanchang, China; The First Affiliated Hospital of Zhengzhou University, Zhengzhou, China; Jiujiang No. 1 People’s Hospital, Jiujiang, China; Zhujiang Hospital of Southern Medical University, Guangzhou, China; and The Second Affiliated Hospital of Guangzhou Medical University, Guangzhou, China), between January 1, 2005, and December 31, 2018. To maximally represent the real-world settings of the CAPD population, no patient was excluded from this study. The study was approved by the Human Ethics Committee of each research centre, consistent with the ethical principles of the Declaration of Helsinki. The data were anonymous, and the need for informed consent was therefore waived.

### 2.2 Data Collection and Definitions

Data on demographics, comorbid conditions, medications, and laboratory values at the start of CAPD were abstracted from medical records by two trained investigators in each centre using uniform and standardized data collection tools: demographic characteristics (age, sex, body mass index [BMI], systolic BP, diastolic BP, 24-hour urine volume, current smoking, and current alcohol consumption); comorbidities (diabetes mellitus, preexisting CVD, hypertension, and hyperlipidaemia); underlying causes of ESRD; medications (calcium channel blockers, beta-blockers, angiotensin-converting enzyme inhibitors/angiotensin II receptor blockers [ACEIs/ARBs], diuretics, statins, and aspirin); and laboratory variables (haemoglobin, serum albumin, serum uric acid, estimated glomerular filtration rate [eGFR], residual renal function [RRF], cholesterol, triglyceride, high-density lipoprotein, low-density lipoprotein, and high-sensitivity C-reactive protein [hs-CRP]). Hypertension was defined as systolic BP >140 mmHg or diastolic BP >90 mmHg or the use of antihypertensive medications according to the 2016 Guidelines for the Management of Renal Hypertension in China [11]. The presence of CVD was defined as coronary heart disease, congestive heart failure, arrhythmias, cerebrovascular disease, or peripheral vascular disease [12]. Current smoking was defined as at least one cigarette a day, and current alcohol consumption was defined as >20 grams of ethanol a day [13]. The estimated glomerular filtration rate was calculated using the Chronic Kidney Disease Epidemiology Collaboration equation [14].

### 2.3 Outcomes and Follow-Up

The primary and secondary outcomes were all-cause and CVD mortality, respectively. If the patients died in any hospital, the exact cause of death was available by death certificates, and if the patients died outside a hospital, experts would reach a consensus on the cause of death, with a comprehensive consideration of current health conditions provided by family members and the medical history and descriptions from each dialysis centre. All participants had conducted CAPD schedules produced by dialysis professionals following the International Standardized Peritoneal Dialysis Guidelines [15] and the patient’s health conditions. All patients were followed up until CAPD cessation, death, the end of an 8-year period, or June 30, 2019. Transferring to haemodialysis, renal transplantation, transferring to other centres, loss of follow-up, and survival after a follow-up period of 8 years or through June 30, 2019 were considered to be censored.

### 2.4 Statistical Analysis

Variables with missing data before the data analysis were imputed using the missForest method, which handles different types of variables [16]. Incidence was calculated as the number of events divided by the total valid observational time at risk, scaled to episodes per 1000 years. Variables are presented as the mean ± standard deviation (SD), median (interquartile range, IQR) or number (%). Patients were divided into four groups: the control group (those without preexisting CVD or hypertension), CVD group (preexisting CVD patients), hypertension group, and CVD plus hypertension group (preexisting CVD patients coexisting with hypertension). Baseline variables were compared by one-way ANOVA or Kruskal‒Wallis tests according to variable distribution (normality tested with the Shapiro‒Wilk test) for quantitative variables and the chi-square test when appropriate for categorical variables among the groups.

We used Kaplan‒Meier curves to investigate the difference in cumulative mortality among the four groups over the observational period. To analyse the association between these interesting comorbidities and mortality, we constructed four Cox proportional hazards regression models adjusted for the following factors: Model 1, unadjusted; Model 2, Model 1 plus age, sex, body mass index, systolic BP, current smoking, current alcohol consumption, diabetes mellitus hyperlipidaemia, and underlying causes of ESRD; Model 3, Model 2 plus medications; and Model 4, Model 3 plus haemoglobin, serum albumin, serum uric acid, RRF, cholesterol, and hs-CRP. In addition, the association was also analysed among subgroups of men, women, diabetes mellitus, nondiabetes mellitus, hyperlipidaemia, and nonhyperlipidaemia. We tested for interactions of sex, diabetes mellitus, and hyperlipidaemia.

### 2.5 Sensitivity Analysis

For all-cause mortality, haemodialysis, renal transplants, loss of follow-up, or transferring to other centres were considered competing risks. When using these competing risks, we evaluated the association between these interesting comorbidities and all-cause mortality using four Fine–Gray competing risk models. Similarly, for CVD mortality, non-CVD mortality, haemodialysis, renal transplants, loss of follow-up, or transfer to other centres were considered competing risks. Second, for adult patients with a short-term period of follow-up, interesting outcomes may not be completely observed, with underreporting of the incidence of mortality. To fully observe outcomes, we further analysed the effect of comorbidities at the start of dialysis on mortality in adult patients with at least a 24-month period of follow-up.

The results of the Cox proportional hazards models and Fine–Gray models are presented as the hazard ratio (HR) and the 95% confidence interval (CI). Statistical analyses were conducted using Stata 15.1 statistical software (StataCorp, College Station, TX, USA). The level of significance was set as 0.05 for all analyses.

## 3. Results

### 3.1 Patient Characteristics and Comorbidities

All 3073 incident CAPD patients from five dialysis centres were included in the present study. All variables with less than 5% missing data were imputed before the data analysis, and there were no missing data for outcomes. Of 3073 patients with a median age of 49.0 (IQR 39.0–61.0), 1780 (57.9%) were men, 1986 (64.7%) had hypertension, 430 (13.9%) had preexisting CVD, and 567 (18.4%) had diabetes mellitus. Compared with the control group, the CVD plus hypertension group tended to be elderly, with higher BMI, systolic BP, haemoglobin, and cholesterol, as well as being more likely to be currently smoking; have diabetes mellitus, hyperlipidaemia, diabetic nephropathy, or hypertensive nephropathy; and taking calcium channel blockers, beta-blockers, diuretics, ACEIs/ARBs, aspirin, and statins but have lower diastolic BP. Compared with patients with hypertension, those with prior CVD were more likely to be older and female, have hyperlipidaemia, and have primary glomerulonephritis and be less likely to be taking calcium channel blockers and ACEIs/ARBs (Table 1).

**Table 1.** **The baseline demographic characteristics, medications, and laboratory parameters among four groups**.

	Overall	Control group	Hypertension group	CVD group	CVD plus hypertension group	*p*-value
Number	3073	1027	1616	60	370	
Age, years	49.0 (39.0–61.0)	45.0 (34.0–56.0)	49.0 (39.0–60.0)	54.0 (44.0–64.0)	62.0 (52.0–70.0)	<0.001
Men, %	1780 (57.9%)	568 (55.3%)	957 (59.2%)	30 (50.0%)	225 (60.8%)	0.080
BMI, kg/$m^{2}$	22.6 ± 7.3	22.0 ± 7.8	22.9 ± 7.6	20.9 ± 5.4	23.2 ± 3.5	<0.001
Systolic BP, mmHg	139.8 ± 25.7	123.1 ± 26.1	153.2 ± 24.5	126.9 ± 26.8	154.6 ± 25.6	<0.001
Diastolic BP, mmHg	83.5 ± 15.8	73.3 ± 15.8	89.6 ± 15.6	74.4 ± 15.7	84.5 ± 15.3	<0.001
24-hour urine volume, mL	800 (500–1200)	800 (440–1200)	800 (500–1200)	900 (400–1262)	800 (450–1200)	0.861
Current smoking (%)	310 (10.1%)	75 (7.3%)	184 (11.4%)	2 (3.3%)	49 (13.2%)	<0.001
Current alcohol consumption (%)	114 (3.7%)	34 (3.3%)	66 (4.1%)	1 (1.7%)	13 (3.5%)	0.608
Diabetes mellitus (%)	567 (18.4%)	67 (6.5%)	318 (19.7%)	7 (11.7%)	175 (47.3%)	<0.001
Hyperlipidemia (%)	567 (18.4%)	140 (13.6%)	282 (17.5%)	20 (33.3%)	125 (33.8%)	<0.001
Underlying causes of ESRD						<0.001
Primary glomerulonephritis (%)	1875 (61.0%)	725 (70.5%)	922 (57.0%)	41 (68.3%)	187 (50.5%)	
Diabetes mellitus (%)	412 (13.4%)	118 (11.5%)	218 (13.5%)	10 (16.7%)	66 (17.8%)	
Hypertension (%)	314 (10.2%)	0 (0.0%)	252 (15.6%)	0 (0.0%)	62 (16.8%)	
Others (%)	472 (15.4%)	184 (17.9%)	224 (13.9%)	9 (15.0%)	55 (14.9%)	
Calcium channel blockers (%)	1626 (52.9%)	0 (0.0%)	1311 (81.1%)	0 (0.0%)	315 (85.1%)	<0.001
Beta blockers (%)	1255(40.8%)	0 (0.0%)	1057 (65.4%)	17 (28.3%)	181 (48.9%)	<0.001
Diuretics (%)	205 (6.7%)	32 (3.1%)	123 (7.6%)	1 (1.7%)	49 (13.2%)	<0.001
ACEIs/ARBs (%)	1042 (33.9%)	0 (0.0%)	849 (52.5%)	19 (31.7%)	173 (46.8%)	<0.001
Aspirin (%)	247 (8.0%)	30 (2.9%)	128 (7.9%)	3 (5.0%)	86 (23.2%)	<0.001
Statins (%)	439 (14.3%)	73 (7.1%)	241 (14.9%)	12 (20.0%)	113 (30.5%)	<0.001
Hemoglobin, g/dL	9.3 ± 2.8	9.2 ± 2.9	9.1 ± 2.8	9.1 ± 2.2	9.8 ± 2.9	<0.001
Serum albumin, g/dL	3.5 ± 0.6	3.5 ± 0.6	3.5 ± 0.5	3.4 ± 0.7	3.5 ± 0.6	0.750
Serum uric acid, mg/dL	6.9 ± 2.3	7.0 ± 2.3	6.9 ± 2.4	6.4 ± 2.2	6.8 ± 2.2	0.184
eGFR, mL/min/1.73 $m^{2}$	6.4 (4.7–8.3)	6.6 (4.7–8.5)	6.4 (4.7–8.2)	6.1 (4.6–8.4)	6.2 (4.7–8.2)	0.415
RRF, mL/min	4.0 (2.0–7.4)	4.1 (2.0–7.4)	4.0 (2.0–7.3)	3.8 (1.9–7.2)	3.9 (1.9–7.3)	0.520
Cholesterol, mg/dL	151 (117–183)	146 (112–179)	153 (118–183)	157 (132–187)	157 (125–187)	0.045
Triglyceride, mg/dL	94 (57–153)	92 (62–149)	95 (53–156)	85 (32–158)	99 (59–153)	0.413
High-density lipoprotein, mg/dL	40 (31–50)	39 (31–51)	40 (32–50)	41 (31–53)	38 (31–48)	0.525
Low-density lipoprotein, mg/dL	82 (48–117)	82 (54–118)	82 (4–116)	89 (37–120)	79 (30–116)	0.861
hs-CRP, mg/L	4.4 (1.9–14.2)	4.1 (1.8–12.5)	4.5 (2.1–14.1)	4.5 (1.7–20.1)	4.4 (1.9–18.9)	0.643

Control group, patients without hypertension or pre-existing CVD. CVD, cardiovascular disease; BMI, body mass index; BP, blood pressure; ACEIs/ARBs, angiotensin-converting enzyme inhibitors/angiotensin II receptor blockers; ESRD, end stage renal disease; eGFR, estimated glomerular filtration rate; RRF, residual renal function; hs-CRP, high-sensitivity C-reactive protein.

### 3.2 Observational Period and Mortality

The median observational period was 33.7 (IQR 15.7–60.9) months. During this period, 571 (18.6%) patients died, with 286 (9.3%) CVD deaths, 59 (1.9%) infection deaths, 10 (0.3%) gastrointestinal bleeding deaths, 17 (0.6%) tumour deaths, 101 (3.3%) other causes of death, and 89 (2.9%) unknown causes of death. In addition, 375 (12.2%) transferred to haemodialysis, 159 (5.2%) received renal transplants, 26 (0.8%) transferred to other dialysis centres, and 106 (3.4%) were lost to follow-up. The number of all-cause mortalities was 143 (38.6%), 15 (25.0%), 293 (18.1%), and 120 (11.7%) in the CVD plus hypertension, CVD, hypertension, and control groups, respectively. The number of CVD mortalities was 76 (20.5%), 7 (11.7%), 147 (9.1%), and 56 (5.5%) in the CVD plus hypertension, CVD, hypertension, and control groups, respectively.

The incidence of all-cause mortality was 55.7/1000 patient-years in the study population, with 27.9/1000 patient-years of CVD mortality incidence (Table 2). The incidence of all-cause mortality was 131.0, 74.4, 56.1, and 32.2/1000 patient-years, and CVD mortality incidence was 69.6, 34.7, 28.2, and 15.0/1000 patient-years among the CVD plus hypertension, CVD, hypertension, and control groups, respectively.

**Table 2.** **All-cause and CVD death incidence**.

	All-cause deaths	CVD deaths	Time at risk (years)	All-cause death incidence (95% CI)	CVD death incidence (95% CI)
Study population	571	286	10252.5	55.7 (53.2–58.6)	27.9 (22.1–34.5)
Control group	120	56	3729.5	32.2 (27.1–38.9)	15.0 (10.3–21.4)
Hypertension group	293	147	5221.9	56.1 (52.5–59.1)	28.2 (21.8–35.2)
Pre-existing CVD group	15	7	201.5	74.4 (69.2–80.6)	34.7 (29.5–43.9)
CVD plus hypertension group	143	76	1091.6	131.0 (128.4–138.7)	69.6 (61.1–78.5)

Incidence was calculated as number of events divided by total valid observational time at risk, scaled to episodes per 1000 years. Control group, patients without HTN and pre-existing CVD. CVD, cardiovascular disease; CI, confidence interval.

### 3.3 Comorbidities and Mortality

**Fig. 1.**
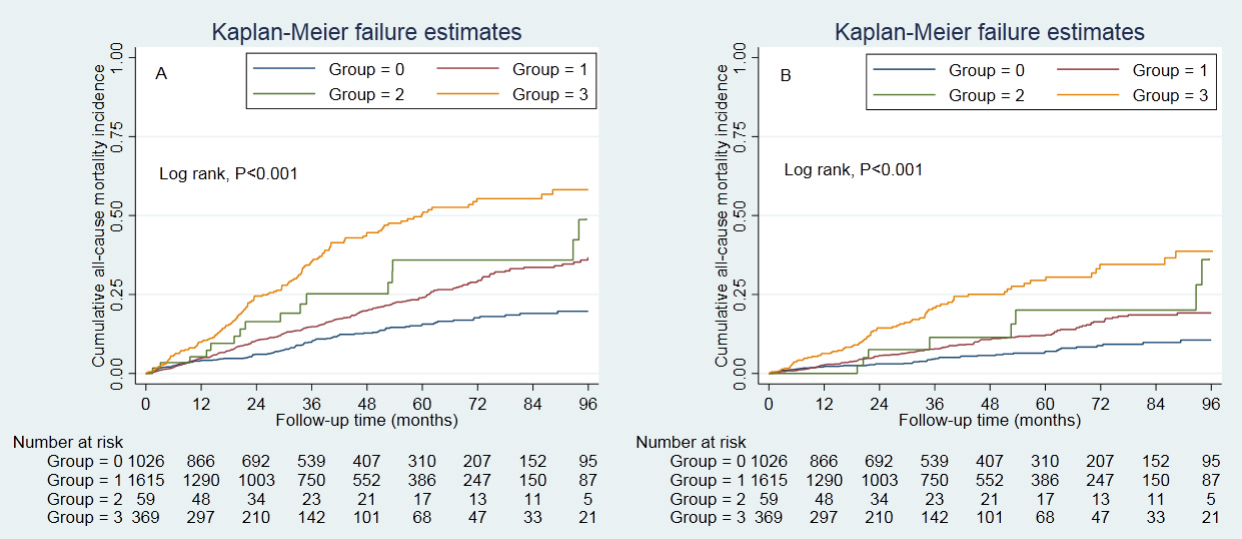
**Cumulative incidence of all-cause and CVD mortality**. (A) presented the cumulative incidence of all-cause mortality, and (B) presented the cumulative incidence of CVD mortality. Cumulative survival was lowest in those with hypertension and prior CVD. Control group, patients without hypertension and prior CVD. Group 0, control group; Group 1, hypertension group; Group 2, prior CVD group; Group 3, hypertension plus prior CVD group. CVD, cardiovascular disease.

**Fig. 2.**
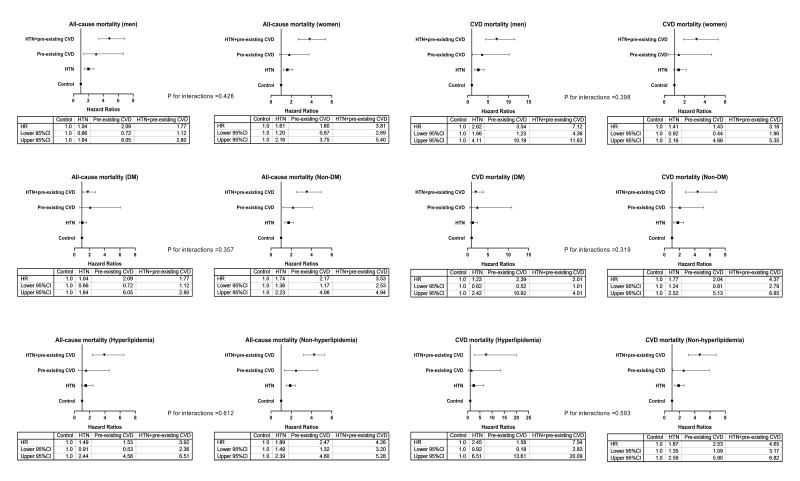
**Adjusted HRs for all-cause and CVD mortality among subgroups**. HRs were adjusted for variables in Model 4, except for the variable of the subgroup. Control group, patients without hypertension and prior CVD. HTN, hypertension; DM, diabetes mellitus; CVD, cardiovascular disease; HR, hazard ratio; CI, confidence interval.

The survival analysis found that the CVD plus hypertension group had greater cumulative all-cause mortality (*p *< 0.001) and CVD mortality (*p *< 0.001) than the control group (Fig. 1). The association between comorbidities and mortality was evaluated by the different Cox proportional hazards regression models (Table 3). When compared with the control group, the CVD plus hypertension, CVD, and hypertension groups had a 3.97-fold (95% CI: 3.06 to 5.15), 2.21-fold (95% CI: 1.29 to 3.79), and 1.83-fold (95% CI: 1.47 to 2.29) higher risk of all-cause mortality and 4.68-fold (95% CI: 3.27 to 6.69), 2.10-fold (95% CI: 0.95 to 4.62), and 1.86-fold (95% CI: 1.36 to 2.54) higher risk for CVD mortality compared with the control group in Model 4, respectively. Similar trends were observed among the subgroups of men, women, diabetes mellitus, nondiabetes mellitus, hyperlipidaemia, and nonhyperlipidaemia (Fig. 2). There was no significant interaction between these subgroup variables and preexisting CVD coexisting with hypertension on all-cause and CVD mortality in the study population. The *p* values for interactions were >0.05 for all subgroups, suggesting that the increased risk of all-cause and cardiovascular mortality associated with interesting comorbidities was evident regardless of these subgroup variables.

**Table 3.** **Adjusted HRs for mortality among different Cox proportional hazards regression models**.

		Model 1		Model 2		Model 3		Model 4	
		HR	95% CI	HR	95% CI	HR	95% CI	HR	95% CI
All-cause mortality									
	Control group	1.0 (ref.)							
	Hypertension group	1.74	1.41 to 2.15	1.77	1.43 to 2.20	1.83	1.47 to 2.27	1.83	1.47 to 2.29
	Pre-existing CVD group	2.35	1.38 to 4.02	2.20	1.28 to 3.76	2.14	1.25 to 3.67	2.21	1.29 to 3.79
	CVD plus hypertension group	4.08	3.20 to 5.20	4.09	3.20 to 5.23	3.91	3.02 to 5.08	3.97	3.06 to 5.15
	*p* for trend		<0.001		<0.001		<0.001		<0.001
CVD mortality									
	Control group	1.0 (ref.)							
	Hypertension group	1.87	1.38 to 2.55	1.93	1.41 to 2.62	1.91	1.39 to 2.59	1.86	1.36 to 2.54
	Pre-existing CVD group	2.33	1.06 to 5.11	2.27	1.04 to 4.99	2.19	1.00 to 4.82	2.10	0.95 to 4.62
	CVD plus hypertension group	4.65	3.29 to 6.57	4.91	3.47 to 6.95	4.67	3.26 to 6.68	4.68	3.27 to 6.69
	*p* for trend		<0.001		<0.001		<0.001		<0.001

Model 1, unadjusted; Model 2, Model 1 plus age, sex, BMI, systolic BP, current smoking, current alcohol consumption, underlying causes of ESRD, diabetes mellitus, and hyperlipidemia; Model 3, Model 2 plus medications; Model 4, Model 3 plus hemoglobin, serum albumin, serum uric acid, RRF, cholesterol, and hs-CRP. Control group, patients without HTN or pre-existing CVD. *p* values for trend across four groups. *p* values for trend were examined by treating groups as a continuous variable in each model. CVD, cardiovascular disease; BMI, body mass index; BP, blood pressure; ESRD, end stage renal disease; RRF, residual renal function; hs-CRP, high-sensitivity C-reactive protein; HR, hazard ratio; CI, confidence interval.

Compared with patients with hypertension (reference), those with prior CVD had a higher risk of all-cause (HR: 1.38, 95% CI: 1.09 to 2.29) and CVD (HR: 1.59, 95% CI: 1.07 to 2.30) mortality (Table 4).

**Table 4.** **Association between prior CVD and mortality (hypertension as a reference) using Cox proportional hazards regression models**.

		Model 1		Model 2		Model 3		Model 4	
		HR	95% CI	HR	95% CI	HR	95% CI	HR	95% CI
All-cause mortality									
	Hypertension group	1.0 (ref.)							
	Prior CVD group	1.52	1.11 to 2.20	1.33	1.04 to 2.36	1.41	1.10 to 2.29	1.38	1.09 to 2.29
CVD mortality									
	Hypertension group	1.0 (ref.)							
	Prior CVD group	1.61	1.15 to 2.24	1.54	1.04 to 2.35	1.60	1.08 to 2.28	1.59	1.07 to 2.30

Model 1, unadjusted; Model 2, Model 1 plus age, sex, BMI, current smoking, current alcohol consumption, underlying causes of ESRD, diabetes mellitus, and hyperlipidemia; Model 3, Model 2 plus medications; Model 4, Model 3 plus hemoglobin, serum albumin, serum uric acid, RRF, and hs-CRP. Control group: patients without hypertension and prior CVD. CVD, cardiovascular disease; BMI, body mass index; ESRD, end stage renal disease; RRF, residual renal function; hs-CRP, high-sensitivity C-reactive protein; HR, hazard ratio; CI, confidence interval.

### 3.4 Sensitivity Analysis

The joint associations showed similar patterns using the Fine–Gray competing risk models (Table 5). The CVD plus hypertension, CVD, and hypertension groups had a 4.15-fold (95% CI: 3.15 to 5.49), 1.99-fold (95% CI: 1.07 to 3.70), and 1.57-fold (95% CI: 1.25 to 1.98) higher risk of all-cause mortality than the control group, respectively. Similarly, compared with the control group, the CVD plus hypertension, CVD, and hypertension groups had a 2.90-fold (95% CI: 1.92 to 4.38), 1.85-fold (95% CI: 0.82 to 4.16), and 1.30-fold (95% CI: 1.08 to 1.79) higher risk of CVD mortality, respectively.

**Table 5.** **Adjusted HRs for mortality among the Fine and Gray competing risk models**.

		Model 1		Model 2		Model 3		Model 4	
		HR	95% CI	HR	95% CI	HR	95% CI	HR	95% CI
All-cause mortality									
	Control group	1.0 (ref.)							
	Hypertension group	1.57	1.27 to 1.95	1.61	1.29 to 2.02	1.62	1.28 to 1.04	1.57	1.25 to 1.98
	Pre-existing CVD group	2.34	1.31 to 4.17	2.23	1.24 to 4.02	2.00	1.09 to 3.75	1.99	1.07 to 3.70
	CVD plus hypertension group	3.52	2.51 to 4.94	4.46	3.45 to 5.78	4.17	3.15 to 5.52	4.15	3.15 to 5.49
	*p* for trend		<0.001		<0.001		<0.001		<0.001
CVD mortality									
	Control group	1.0 (ref.)							
	Hypertension group	1.73	1.28 to 2.33	1.75	1.27 to 2.41	1.73	1.25 to 2.40	1.30	1.08 to 1.79
	Pre-existing CVD group	2.18	1.09 to 4.37	2.09	0.88 to 4.95	1.95	0.88 to 4.43	1.85	0.82 to 4.16
	CVD plus hypertension group	4.40	2.55 to 7.58	3.08	2.15 to 4.42	2.97	1.97 to 4.49	2.90	1.92 to 4.38
	*p* for trend		<0.001		<0.001		<0.001		<0.001

Model 1, unadjusted; Model 2, Model 1 plus age, sex, BMI, systolic BP, current smoking, current alcohol consumption, underlying causes of ESRD, diabetes mellitus, and hyperlipidemia; Model 3, Model 2 plus medications; Model 4, Model 3 plus hemoglobin, serum albumin, serum uric acid, RRF, cholesterol, and hs-CRP. Control group, patients without HTN or pre-existing CVD. *p* values for trend across four groups. *p* values for trend were examined by treating groups as a continuous variable in each model. CVD, cardiovascular disease; BMI, body mass index; BP, blood pressure; ESRD, end stage renal disease; RRF, residual renal function; hs-CRP, high-sensitivity C-reactive protein; HR, hazard ratio; CI, confidence interval.

A total of 42 (1.4%) patients aged <18 years at the start of dialysis were excluded, with six deaths at the end of the study. By the end of the study, 810 (26.3%) adult patients were followed up for less than 24 months, and 282 (9.2%) adult patients survived for less than 24 months. The remaining 1939 (63.1%) adult patients were followed up for at least 24 months, with 283 (14.6%) all-cause mortality and 135 (7.0%) CVD mortality. We found that compared with the control group, the CVD plus hypertension, CVD, and hypertension groups had a 3.92-fold (95% CI: 2.76 to 5.57), 1.99-fold (95% CI: 1.47 to 2.71), and 1.58-fold (95% CI: 1.17 to 2.13) higher risk of all-cause mortality and 4.47-fold (95% CI: 2.68 to 7.46), 1.78-fold (95% CI: 0.54 to 5.90), and 1.48-fold (95% CI: 1.28 to 1.71) higher risk of CVD mortality in Cox regression Model 4, respectively, among adult adults with at least a 24-month follow-up period (data not shown).

## 4. Discussion

We found that preexisting CVD and hypertension at the start of PD were additive prognostic utilities for mortality, and preexisting CVD was more strongly associated with mortality than hypertension. Our findings were robust because similar trends were observed by the competing risk analysis and among subgroups as well as in those with at least a 24-month period of follow-up.

In our study, the prevalence of preexisting CVD coexisting with hypertension was 12.0%. However, to date, no study has reported the predictors for preexisting CVD coexisting with hypertension. We first reported that elderly age; diabetes mellitus; hyperlipidaemia; higher systolic BP, diastolic BP, and cholesterol; and lower low-density cholesterol were independently associated with a higher risk of preexisting CVD coexisting with hypertension. Among these predictors for preexisting CVD coexisting with hypertension, diabetes mellitus was the strongest predictor, followed by hyperlipidaemia. Interestingly, lower levels of low-density cholesterol were associated with a higher risk of preexisting CVD coexisting with hypertension, which seemed to contradict clinical knowledge. Actually, this evidence may be in line with clinical knowledge. In clinical setting, the cholesterol targets in patients with pre-existing CVD are lower than primary prevention patients. In fact, the recruited patients have already experienced a cardiovascular disease and did not develop it during follow up. Moreover, we would like to underline that statin use for secondary prevention in dialysis patients is still debated, though it has been reported a reduced incidence in major adverse cardiovascular events in a recent study [17]. The reason may be that preexisting CVD coexisting with hypertension had received extensive lipid management, resulting in lower levels of low-density cholesterol. More importantly, we found that preexisting CVD and hypertension were additive risk factors for mortality, and preexisting CVD was more strongly associated with mortality than hypertension. Similar findings were observed by the competing risk analysis and among subgroups as well as in patients with at least a 24-month follow-up. These findings suggest that preexisting CVD coexisting with hypertension is associated with the highest risk of mortality, followed by preexisting CVD and hypertension.

Hypertension is highly prevalent and plays a significant role in the mortality of dialysis patients [16]. Previous observational studies over the past decade have confirmed the “U-shaped” or “reverse J-shaped” relationship between BP and mortality in dialysis patients [18, 19, 20, 21]. In contrast, a direct linear association between systolic BP outside the unit and all-cause mortality was observed (HR: 1.26 for each 10 mmHg higher systolic BP; 95% CI: 1.14 to 1.40) [22]. However, few studies have focused on the association between hypertension, as a comorbidity, and mortality in dialysis patients. In the present study, after adjusting for confounding factors, hypertension patients had 1.83-fold higher all-cause mortality and 1.87-fold higher CVD mortality compared with patients without hypertension and preexisting CVD, and similar findings were found by the sensitivity analysis and in the subgroup analysis. Meanwhile, a study of 107,922 dialysis patients from the United States evaluated the association between dialysis modality and mortality, with 26.0% of new ESRD patients having coronary artery disease [4]. The HR of death was significantly greater for patients with coronary artery disease than for those without these conditions at ESRD onset CAD (HR: 1.11, 95% CI: 1.08 to 1.14). We previously conducted a study of 1068 Chinese CAPD patients, where 30.8% were preexisting CVD patients from another dialysis centre [10]. We reported that 7.0% of prior stroke CAPD patients (n = 75) had a 1.82-fold higher risk of all-cause mortality than patients without this condition [10]. In the present study, when using patients without hypertension and preexisting CVD as a reference, patients with only preexisting CVD had a 2.21-fold higher all-cause mortality and 2.10-fold higher CVD mortality. Additionally, similar results were observed by the sensitivity analysis and in the subgroup analysis. The HR of preexisting CVD for all-cause mortality was significantly higher than the HR of 1.11 in the aforementioned study with a 2-year follow-up period [4]. The disparities in these findings may be due to (1) different ethnicities, (2) different sample sizes, and (3) different follow-up durations. Our findings mentioned above indicated that patients with only preexisting CVD were at higher risk of all-cause and CVD mortality than those with only hypertension than patients without hypertension and preexisting CVD.

In the present study, baseline demographic characteristics, medications, and laboratory parameters were unmatched among the four patient categories. Compared with the other three patient categories, patients with preexisting CVD and hypertension were more likely to be elderly; have higher percentiles of current smoking and diabetes mellitus; be taking medications; have hyperlipidaemia; have higher levels of systolic BP, haemoglobin, and cholesterol; and have lower levels of diastolic BP. Elderly age, current smoking, and diabetes mellitus have an adverse effect on the prognosis of dialysis patients [23, 24, 25, 26]. These unmatched variables at baseline among the four patient categories may affect the association between preexisting CVD coexisting with hypertension, preexisting CVD, hypertension, and mortality. Thus, although adjusting baseline variables, unmatched variables at baseline among patient subcategories may affect our findings, and we should in the near future conduct a cohort study with well-balanced variables at baseline to validate the association between these interesting comorbidities and mortality among CAPD patients. Additionallly, the role of type 2 diabetes is major compared to hypertension. It should be noted that hypertension is part of metabolic syndrome, which exposes to increased cardiovascular disease. The culmination of the metabolic syndrome is represented by type 2 diabetes, which have been addressed as one of the major factors involved in increased mortality in this subgroup of patients. Moreover, diabetes is also responsible per se of cardiac modifications, the so-called diabetic cardiomyopathy [27].

The strengths of this study included a large sample size, a population from five dialysis centres, and detailed evaluation and adjustment for all-cause and CVD risk factors for real-world data. Several limitations should be considered. First, this was a retrospective study with potential unaccounted-for confounding factors. As all patients were included, patients who missed baseline covariates, with expected limited life expectancy (such as coexisting with malignant disease) and coexisted with undetected asymptomatic CVD increased the risk of selection bias. After adjusting for baseline variables, we did not draw conclusions about the potential causal relationship between comorbidities and mortality. Nonetheless, fluctuations in HRs among Models 2, 3, and in the fully adjusted model were less than 10%, suggesting that the three models were stable and reliable for predicting outcomes [28]. Second, one challenge was the definition of hypertension. The optimal method for diagnosing hypertension in peritoneal dialysis patients is an area of controversy [29]. A recent study reported that similar to the general population, ambulatory BP monitoring is the gold standard method in the management of hypertension in PD patients [30]. In our study, given the effect of ethnicity on Chinese CAPD population settings, the diagnosis of hypertension was based on the 2016 Guidelines for the Management of Renal Hypertension in China [11]. Nontheless, blood pressure medications may be used for indications other than hypertension which may increase bias (e.g., frusemide use in fluid retention as opposed to hypertension). Third, although we tried to reach a consensus on causes of death, the causes of death were not identified in 89 (2.9%) patients, which may affect the association between these interesting comorbidities and CVD mortality. Last, although we tried to increase the generalizability of CAPD population settings with broad inclusion criteria, all patients were from China, suggesting that our findings may lack generalization to other ethnic populations.

## 5. Conclusions

In conclusion, preexisting CVD and hypertension at the start of CAPD may provide additive prognostic information for mortality in this setting. Additionally, preexisting CVD was more strongly associated with mortality. Our findings suggested that a combined assessment of preexisting CVD and hypertension compared with a separate assessment of the two comorbidities further improved the risk stratification of CAPD patients at risk of mortality.

## Data Availability

The datasets used and/or analyzed during the current study are available from the corresponding author on reasonable request.
